# Death from Chloroform

**Published:** 1868-06

**Authors:** B. F. Brown

**Affiliations:** Oneida, Knox Co., Ill.


### DEATH FROM CHLOROFORM.

BY B. F. BROWN, M.D., Oneida, Knox Co., Ill.

Mrs. Elizabeth Hammon, aged 35, mother of three children, apparently a healthy woman, went to a dentist (Dr. McDowell) April 16, 1868, for the purpose of having some teeth extracted. Thinking she could not endure the pain, she requested the Doctor to administer chloroform; and, as he had given it to her once before (about six months since) without any bad effect, he consented to administer it again, and pouring about two drachms of chloroform upon a sponge, held it a short distance from her face. After she had made three or four inspirations her respiration ceased; he felt for the pulse and found she had none. Artificial respiration was commenced at once, and stimulating applications applied over the heart and to the extremities, but to no effect. She made but two or three efforts at inspiration after he first noticed something was wrong. An autopsy could not be obtained.

